# Ciprofloxacin Derivatives Affect Parasite Cell Division and Increase the Survival of Mice Infected with *Toxoplasma gondii*

**DOI:** 10.1371/journal.pone.0125705

**Published:** 2015-05-07

**Authors:** Erica S. Martins-Duarte, Faustine Dubar, Philippe Lawton, Cristiane França da Silva, Maria de Nazaré C. Soeiro, Wanderley de Souza, Christophe Biot, Rossiane C. Vommaro

**Affiliations:** 1 Universidade Federal do Rio de Janeiro—Instituto de Biofísica Carlos Chagas Filho, Rio de Janeiro, Brazil; 2 Instituto Nacional de Ciência e Tecnologia em Biologia Estrutural e Bioimagens, Rio de Janeiro, Brazil; 3 Université de Lille1—Unité de Glycobiologie Structurale et Fonctionnelle, UGSF, F-59650 Villeneuve d'Ascq, France; 4 CNRS, UMR 8576, F-59650 Villeneuve d'Ascq, France; 5 Université de Lyon, Université Claude-Bernard Lyon1, ISPB-Faculté de Pharmacie, Lyon, France; 6 Laboratório de Biologia Celular do Instituto Oswaldo Cruz/Fiocruz, Rio de Janeiro, Brazil; 7 Instituto Nacional de Metrologia, Qualidade e Tecnologia—Inmetro, Rio de Janeiro, Brazil; Univ. Georgia, UNITED STATES

## Abstract

Toxoplasmosis, caused by the protozoan *Toxoplasma gondii*, is a worldwide disease whose clinical manifestations include encephalitis and congenital malformations in newborns. Previously, we described the synthesis of new ethyl-ester derivatives of the antibiotic ciprofloxacin with ~40-fold increased activity against *T*. *gondii in vitro*, compared with the original compound. Cipro derivatives are expected to target the parasite’s DNA gyrase complex in the apicoplast. The activity of these compounds *in vivo*, as well as their mode of action, remained thus far uncharacterized. Here, we examined the activity of the Cipro derivatives *in vivo*, in a model of acute murine toxoplasmosis. In addition, we investigated the cellular effects *T*. *gondii* tachyzoites *in vitro*, by immunofluorescence and transmission electron microscopy (TEM). When compared with Cipro treatment, 7-day treatments with Cipro derivatives increased mouse survival significantly, with 13–25% of mice surviving for up to 60 days post-infection (vs. complete lethality 10 days post-infection, with Cipro treatment). Light microscopy examination early (6 and 24h) post-infection revealed that 6-h treatments with Cipro derivatives inhibited the initial event of parasite cell division inside host cells, in an irreversible manner. By TEM and immunofluorescence, the main cellular effects observed after treatment with Cipro derivatives and Cipro were cell scission inhibition - with the appearance of ‘tethered’ parasites – malformation of the inner membrane complex, and apicoplast enlargement and missegregation. Interestingly, tethered daughter cells resulting from Cipro derivatives, and also Cipro, treatment did not show MORN1 cap or centrocone localization. The biological activity of Cipro derivatives against *C*. *parvum*, an apicomplexan species that lacks the apicoplast, is, approximately, 50 fold lower than that in *T*. *gondii* tachyzoites, supporting that these compounds targets the apicoplast. Our results show that Cipro derivatives improved the survival of mice acutely infected with *T*. *gondii* and inhibited parasite replication early in the first cycle of infection *in vitro*, highlighting their therapeutic potential for the treatment of toxoplasmosis.

## Introduction

*Toxoplasma gondii*, the causative agent of toxoplasmosis, is a globally spread parasite infecting approximately one third of the human population. Toxoplasmosis has a broad spectrum of clinical presentations. Infection of the central nervous system by this parasite is associated to encephalitis and eye disease. Indeed, the vertical transmission during pregnancy can lead to abortion and congenital malformations in the newborn [1].Whereas infections by this parasite are usually asymptomatic in most individuals, *T*. *gondii* is an important opportunistic pathogen with high mortality and morbidity in immunocompromised patients [2]. Indeed, ocular toxoplasmosis rates can be extremely high, even in immunocompetent individuals, especially in South American countries [2–4].

Despite the worldwide prevalence of this potentially serious medical condition, toxoplasmosis treatment is limited to a small number of drugs that are associated with numerous side effects. The combination of pyrimethamine with sulfadiazine is the first choice of treatment for toxoplasmosis; however, patients often do not tolerate sulfadiazine, and long-term treatment (4–6 weeks) with this drug is commonly associated with gastrointestinal disorders that lead to treatment discontinuation. In patients with intolerance to sulfadiazine, pyrimethamine is combined with clindamycin or atovaquone, which also cause gastrointestinal disorders [5,6]. Overall, it is clear that the development of alternative or replacement treatments for toxoplasmosis is vital for improving disease treatment and control.

The discovery of a ‘relic’ chloroplast (apicoplast) in apicomplexan parasites – a group that includes *T*. *gondii* and also *Plasmodium spp*., the causative agents of malaria—brought new possibilities for the identification of novel targets for therapy against these parasites [7, 8]. Although photosynthetic function was lost during apicoplast evolution, this organelle still harbors many metabolic pathways of prokaryotic origin and whose functions are essential for parasite survival [9, 10]. Due to their prokaryotic origins, the apicoplast pathways represent interesting targets for the development of specific anti-parasitic compounds with limited toxicity to host cell pathways of eukaryotic origin. The chemotherapeutic potential of the metabolic activities operating in the apicoplast is well recognized [8, 11–13], the antibiotics clindamycin and azithromycin, inhibitors of apicoplast protein synthesis, are already in clinical use as a second-line therapy for toxoplasmosis treatment [5].

Among apicoplast pathways, DNA replication is an important potential chemotherapeutic target. Fluoroquinolones are known DNA replication inhibitors that target prokaryotic type II topoisomerases (DNA gyrase and topoisomerase IV) [14] These antibiotics have a broad spectrum of activity against various pathogens, including bacteria, mycoplasma, and protozoan parasites [15, 16]. They are generally well tolerated by most patients, and are commonly used in the clinic [17].

DNA gyrases introduce negative supercoiling into the DNA strand during DNA replication and transcription. The DNA gyrase enzymatic complex is formed of two copies of both A and B subunits (A_2_B_2_); subunits A catalyze DNA double-strand breaks, while subunits B have ATPase function [14]. Homologues of DNA gyrase subunits A and B genes are found in the nuclear genome of apicomplexan parasites (probably as a result of gene transfer from the organelle’s ancestral genome, during apicoplast evolution), and their protein products are targeted exclusively to the apicoplast [18]. Pharmacological inhibition of subunits A and B of the apicoplast’s DNA gyrase – by fluoroquinolone and novobiocin, respectively—inhibit apicoplast genome replication and affect parasite viability, validating this enzyme as a potential drug target in apicomplexans [8, 19].

In a previous work, we described the synthesis and *in vitro* tests against *P*. *falciparum* and *T*. *gondii* of novel ester prodrugs of ciprofloxacin (Cipro), a known fluorquinolone [20]. Chemical modifications of the reference compound yielded, on average, a 40-fold increase in the anti-parasitic activity compared with the original molecule, and Cipro derivatives had low toxicity against mammalian cells (murine splenocytes and the LLCMK_2_ epithelial cell line) [20].

Among the ester prodrugs of Cipro tested against *T*. *gondii in vitro*, compounds 2 (pro-drug), 4 (phenyl substituent) and 5 (adamantanyl substituent) were particularly potent, with IC_50_ values in the nanomolar or low micromolar range (0.42 μM and 0.46 μM, for compounds 2 and 5, respectively, and 1.24 μM for compound 4, after 48 h of treatment) [20]. The presence of ethyl esters on the carboxyl of Cipro contributed to increase the activity of these derivatives compared with that of the original compound [20]. Esterification of Cipro into prodrug compounds is expected to make the original molecule more lipophilic, favoring the diffusion across biological membranes—including the multiple membranes of *T*. *gondii*, and also those of the apicoplast, which harbors the likely target of Cipro derivatives (DNA gyrase).

Although the Cipro derivatives synthesized and described by us previously are expected to target the apicoplast’s DNA gyrase complex, their cellular mode of action in apicomplexans has not been examined. Also, these drugs were not tested *in vivo* against infections by apicomplexan parasites.

In the present study, we evaluated the activities of compounds 2, 4 and 5 against *T*. *gondii* infection *in vivo*, in a murine model of acute toxoplasmosis. Also, we characterized the cellular effects of these compounds – henceforth referred to as Et-Cipro (compound 2), Ph-Cipro (compound 4), and Adam-Cipro (compound 5)—to elucidate their mode of action in *T*. *gondii*. We show that Et-cipro and Adam-cipro treatment increased mouse survival compared with Cipro treatment, disrupted parasite cell division and affected apicoplast segregation. Interestingly, Cipro derivatives could not inhibit the proliferation of *Cryptosporidium parvum*, an apicomplexan species that lacks the apicoplast.

## Materials and Methods

### Ethical Statement

The experimental protocols for animal use in this study were approved by the institutional Ethics Committee for Animal Use (CEUA, IBCCF, UFRJ: approval IDs 208-09/16 and 206-09/16; CEUA Fiocruz—LW-16/13), and are in agreement with the Brazilian federal law (11.794/2008, Decreto n° 6.899/2009).

### Parasites and Mice

In this study, we used tachyzoite forms the RH strain of *Toxoplasma gondii*, obtained from the peritoneal cavity of mice 2 days after infection [21]. Four to six-week-old female Swiss mice (18–22 g) were used for the *in vivo* experiments. Drinking water and food were given *ad libitum*.

### *In vivo* toxicity analysis

Acute toxicity analysis was performed using non-infected female Swiss mice (19–21 g). Mice were administrated a single oral dose of Et-Cipro, Ph-Cipro or Adam-Cipro (25, 50, 100, or 200 mg/kg/day) and monitored for a period of 48 hours for the appearance of toxic and sub-toxic symptoms (weight body loss and animal behavior alterations). During the toxicity analysis no animal has died, then after the 48h period of observation after drug administration mice were anesthetized with CO_2_ and blood was collected by cardiac puncture to determine the serum levels of urea and creatinine kinase at CECAL/Fiocruz platform (Ortho Clinical-Johnson & Johnson), as reported previously [22].

To determine compound efficacy against *T*. *gondii in vivo*, female Swiss mice were infected intraperitoneally (i.p.) with 5x10^3^ *T*. *gondii* tachyzoites and treated with test compounds from 24 h post-infection. Groups of 3–4 mice were housed per cage and arbitrarily assigned to one of the following treatment groups: Cipro, Et-Cipro, Ph-Cipro or Adam-Cipro (50–150 mg/kg/day), or untreated (i.e., treated with vehicle, polyethylene glycol/PEG). Mice were treated once daily for 7 days, by oral gavage, and mouse mortality was monitored once a day for a period of 60 days. During survival studies, mice were not inflicted to any suffering condition and mice presenting morbidity symptoms (shivering, ruffled hair and immobility) were euthanized by CO_2_ asphyxiation to minimize animal suffering, and then mortality was scored. Survival curves were calculated using the Kaplan and Meier method, and compared using the log-rank (Mantel-Cox) test, in GraphPad Prism 5.0 (GraphPad Software Inc.) and *P<0*.*05* was considered statistically significant. The following numbers of mice were used in this study: in untreated groups, n = 10 (Cipro control) or n = 14/15 (Et-Cipro, Ph-Cipro and Adam-Cipro controls), in 3–4 groups; in Cipro groups, n = 11 (50 and 100 mg; 3 groups) and n = 8 (150 mg Cipro; 2 groups); in Et-Cipro groups, n = 11 (50 and 100 mg; 3 groups); in Ph-Cipro groups, n = 8 (50 and 100 mg; 2 groups) and n = 11 (150 mg; 3 groups); and in Adam-Cipro groups, n = 3 (50 mg; 1group) and n = 12 (100 mg; 3 groups).

### Drug treatments *in vitro*

For the treatment of *T*. *gondii* tachyzoites with Cipro derivatives *in vitro*, 5x10^5^ LLC-MK_2_ cells (ATCC CCL7, Rockville, MD/EUA) in RPMI medium were plated on coverslips inside 24-well tissue culture plates, and allowed to settle at 37°C (5% CO_2_) for ~24h. Then, cells were infected with parasites (freshly-harvested from infected mice) at a 10:1 ratio of parasites to host cells. Tachyzoites were allowed to interact with host cells for 1 h, cell monolayers were washed three times with medium (to remove extracellular parasites) and then treated with different concentrations of Cipro (20–50μM), Et-Cipro (0.5–5 μM), or Adam-Cipro (0.5–5 μM), for 6 h at 37°C. Then, coverslips were fixed with Bouin and stained with panoptic (solutions 2 and 3 of the ‘Panotico rápido’ kit, from Laborclin Ltda., Paraná, Brazil). Alternatively, cultures were washed three times with 0.5 ml of medium (to remove test compounds) and incubated for an additional 18 h in medium without drugs, before fixing and staining. The number of parasites per vacuole was scored by direct light microscopy examination. Three independent experiments were performed for each treatment condition, and the results were analyzed by two-way ANOVA (using GraphPad Prism 5.0).

For plaque assay, 6-well tissue culture plates were seeded with human foreskin fibroblasts (HFF; ATCC). HFF was grown in high glucose Dulbeco’s Modified Eagle Medium supplemented with 10% FBS, 2mM L-glutamine, 100 U/ml penicillin, and 100 μg/ml streptomycin and maintained at 37°C with 5% CO_2_. After cultures reached confluence each well was infected with 10^4^ of *T*. *gondii*. Infected cultures were treated for 10 days with Cipro derivatives, Cipro or left untreated.

### Immunofluorescence microscopy

For immunofluorescence assays, LLC-MK_2_ cells infected with tachyzoites at a 10:1 ratio of parasites to host cells were treated with Et-Cipro, Adam-Cipro and Ph-Cipro compounds for 24 h. For Cipro assay, cells were infected in a ratio of 5:1 parasites per host cell and treated for 24, 48 and 72h. After treatment, infected cells were fixed in 3.7% freshly-prepared formaldehyde, permeabilized with 0.5% Triton X-100 for 15 min, and blocked with 3% bovine serum albumin (BSA) in PBS at pH 7.4 and room temperature for 1 h. All antibody incubations were performed in 3% BSA in PBS, for 1h, at room temperature. The following primary antibodies were used: anti-SAG1 (kindly provided by Dr. John Boothroyd, Stanford University School of Medicine, USA), at a dilution of 1:1000; anti-HSP60 and anti-Morn1 (kindly provided by Dr. Boris Striepen, University of Georgia, USA), diluted to 1:2000 and 1:100, respectively; and anti-IMC1 (kindly provided by Dr. Gary Ward, University of Vermont, USA), diluted to 1:1000. The secondary antibodies used here were anti-mouse/rabbit coupled to Alexa 488 or 546 (Molecular Probes). Sytox green (Molecular Probes) and DAPI (Sigma-Aldrich) were used to label the DNA. After labeling, the coverslips were mounted onto slides using Prolong gold (Life Technologies), and samples were examined on a TCSSP5 Leica or Zeiss LSM710 laser scanning confocal microscopes.

### Transmission electron microscopy (TEM)

For TEM, LLC-MK_2_ cultures were infected with tachyzoites as described above and then treated with 1 and 5 μM Et-Cipro and Adam-Cipro for 24 and 48 h, 20 μM Cipro for 48 and 72h or left untreated (control). After treatment, cells were fixed in 2.5% glutaraldehyde in 0.1 M sodium cacodylate buffer (pH 7.4), and post-fixed for 45 min (and in the dark) in 1% osmium tetroxide, 1.25% potassium ferrocyanide and 5 mM CaCl_2_, in 0.1 M sodium cacodylate buffer (pH 7.4). Samples were dehydrated in acetone solutions of increasing concentrations (30–100%) and embedded in PolyBed (Polyscience Inc., Warrington, PA, USA). Ultrathin sections were stained with uranyl acetate and lead citrate, and then observed in a Zeiss 900 Electron Microscope (Carl Zeiss, Inc.) or in a Jeol 1200 EX electron microscope (Jeol LTD, Tokyo, Japan).

### *Cryptosporidium parvum in vitro* assay

*C*. *parvum* oocysts obtained from infected neonatal calves (Institut National de la Recherche Agronomique-INRA, Nouzilly, France) were purified as previously described [23] and stored in PBS at 4°C. Madin Darby bovine kidney cell line (MDBK; ECACC # 88081201, Sophia Antipolis, France) was cultured at 37°C in a 5% CO_2_ moist atmosphere in growth medium: RPMI 1640 (Sigma, L’Isle d’Abeau, France) supplemented with 25 mM HEPES (Sigma H-3375), 200 U/ml of penicillin, 200 μg/ml of streptomycin (Sigma P-0781) and 10% fetal calf serum (FCS; Dutscher, Brumath, France).

Prior to infection, MDBK cells were trypsinized and seeded in just one well of a 24-well plate in 2 ml of RPMI 1640 with 1% FCS. Aseptic oocysts were prepared for excystation by incubation in RPMI-1640, pH 2 (for 30 min at 37°C). Then, oocytes were washed in RPMI 1640 with 1% FCS and allowed to interact with MDBK cells for 1.5 h at 37°C in a 5% CO_2_ atmosphere_._ After 3 washes by centrifugation at 160 *g* in RPMI 1640 to remove non-excysted oocysts and shells, infected cells were plated in 24-well plates containing glass coverslips, in 500 μl/well of RPMI/1%FCS, and allowed to settle for 24 h. The infected cells were subsequently washed 3 times with RPMI 1640 and allowed to grow for a further 5 h (in 500 μl/well of RPMI/1% FCS). 500 μl of medium containing twice-concentrated Cipro derivatives were then added in triplicates in an equal volume of growth medium and left for another 24 h. Cells were fixed in 100% methanol and stained with 20% Giemsa and 1% Alcian blue as previously described [24], and the effects against sporozoite proliferation were evaluated by examining at least 1000 cells/coverslip in a light microscope, using an oil immersion X100 objective.

## Results

### Cipro derivatives increased the survival of mice acutely infected with *T*. *gondii*

In a previous study, we showed that new prodrugs derivatives of the known antibiotic ciprofloxacin were at least 40 fold more active than the original molecule against *T*. *gondii* tachyzoites, the form of the parasite that causes acute disease [20]. In preparation for *in vivo* testing of ciprofloxacin derivatives against *T*. *gondii* infection in mice, we performed an acute toxicity analysis of the test compounds using a ‘single-dose assay’, where uninfected mice were administered with a single oral dose of 25–200mg/kg of Cipro derivatives, and then monitored for side effects for up to 48 h. During the toxicity assay, no animal has died and no alterations in mouse behavior or body weight gain were observed 48 h after drug administration (Table 1). Biochemical assays showed that serum urea levels increased 48 hours after treatment with Cipro derivatives; however, this was also observed in vehicle-treated animals. Mice treated with higher doses of Et-Cipro and Adam-Cipro showed normal serum levels of creatinine kinase, but animals treated with 200 mg/kg of Ph-Cipro had slightly altered serum levels of this enzyme.

**Table 1:** Toxicity of ciprofloxacin derivatives in mice.

Compound dose (mg/kg)	Mouse weight (g)		Serum urea^a^	Serum creatinine kinase^b^
	Initial	After 48 h		
Vehicle	20.15	21.28	53.0	ND
**Et-Cipro** 25	19.87	22.25	55.8	1727
50	19.15	20.92	51.2	182
100	20.71	22.27	55.1	578
200	20.0	22.17	49.3	583
Vehicle	19.30	21.91	48.0	420
**Adam-Cipro** 25	20.65	23.10	ND	ND
50	20.80	22.12	ND	ND
100	20.45	23.50	ND	ND
200	21.11	23.28	51.5	467
Vehicle	19.49	22.94	46.7	563
**Ph-Cipro** 25	21.21	23.85	49.2	ND
50	19.30	22.72	50.6	506
100	19.21	21.94	52.8	ND
200	19.93	22.20	46.2	1541

Swiss mice were administrated with a single oral dose of different ciprofloxacin derivatives, and monitored for 48h.

Serum levels of urea and creatinine kinase were measured in blood samples harvested 40h after drug administration. The following reference values (mg/kg) were used:

^a^18–29 mg/dL

^b^, ≤ 1070 U/L.

Swiss mice were administrated with a single oral dose of different ciprofloxacin derivatives, and monitored for 48h.

**Fig 1:**
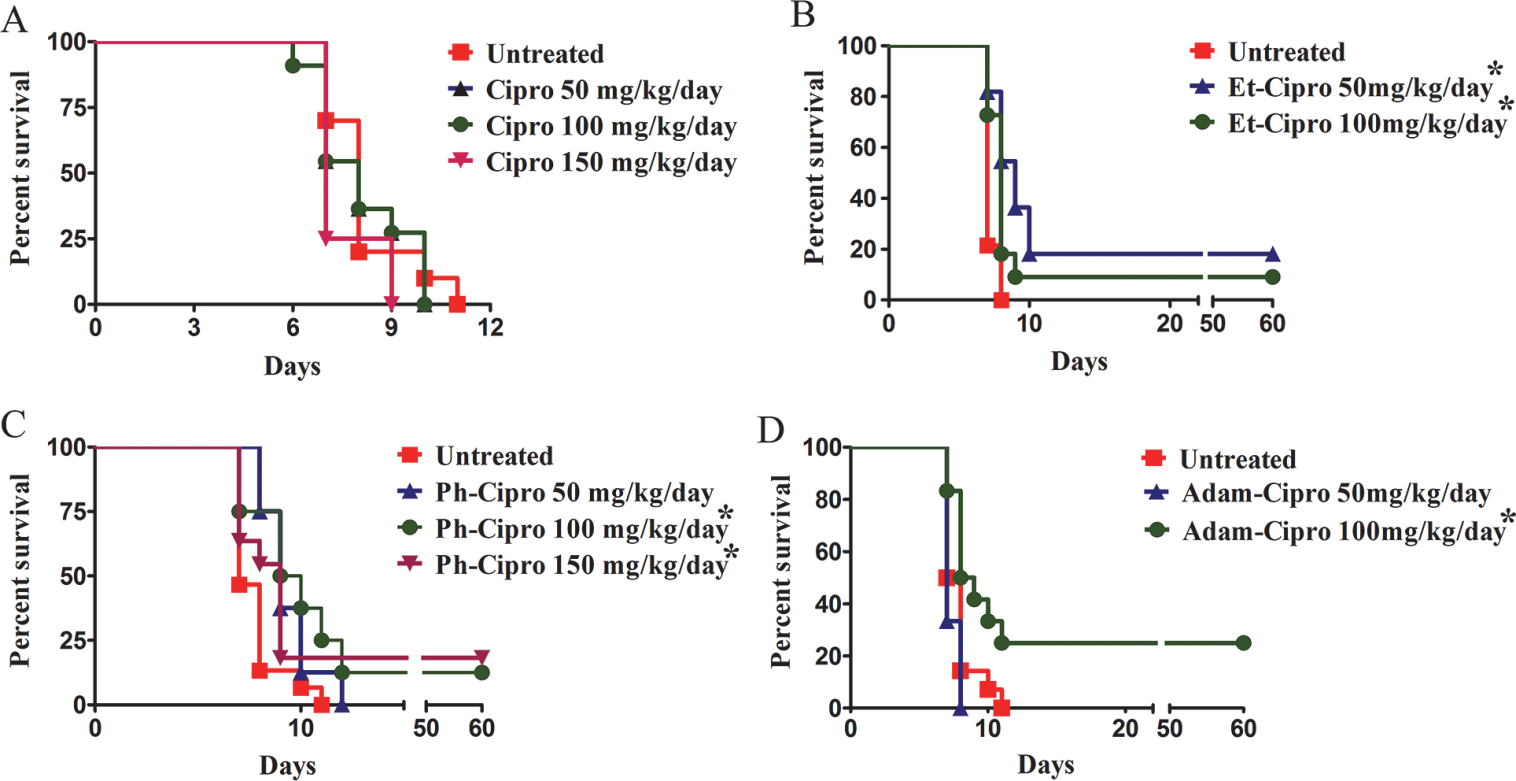
Effect of 7-day treatment with different doses of Cipro derivatives on the survival mice. Swiss Webster mice were infected i.p. with 5x10^3^ tachyzoites of *T*. *gondii* (RH strain) one day prior to the start of treatment. Results were evaluated by the Kaplan-Meier product limit method, and compared using the log-rank (Mantel-Cox) test. * *P*<0.05 vs. untreated controls. The numbers of treated mice in each group were: untreated, 10 (A), 14 (B and D) and 15 (C); 50 and 100 mg Cipro, 11 (three groups each); 150 mg Cipro, 8 (two groups); 50 and 100 mg Et-Cipro, 11 (three groups); 50 and 100 mg Ph-Cipro, 8 (two groups); 150 mg Ph-Cipro, 11 (three groups); 50 mg Adam-Cipro, 3 (one group); 100 mg Adam-Cipro, 12 (three groups).

Treatment of mice infected with *T*. *gondii* tachyzoites with non-toxic doses of Cipro derivatives for 7 days resulted in increased mouse survival (Fig 1A–1D). All mice treated with Cipro died by day 10 post-infection (Fig 1A). In contrast, significantly increased survival of *T*. *gondii*-infected mice was observed after treatment with 50 mg/kg/day Et-Cipro (18%; *P* < 0.05; Fig 1B) or 100 or 150 mg/kg/day Ph-Cipro (13 and 18%, respectively; *P*<0.05; Fig 1C), or 100 mg/kg/day Adam-Cipro (25%; *P*<0.05; Fig 1D). These mice were still alive 60 days post-infection (Fig 1). Brain examination of 60-days survived mice did not show encysted parasites (presence of chronic infection) (data not shown).

### Cipro derivatives inhibit *T*. *gondii* cell division early during infection

**Fig 2:**
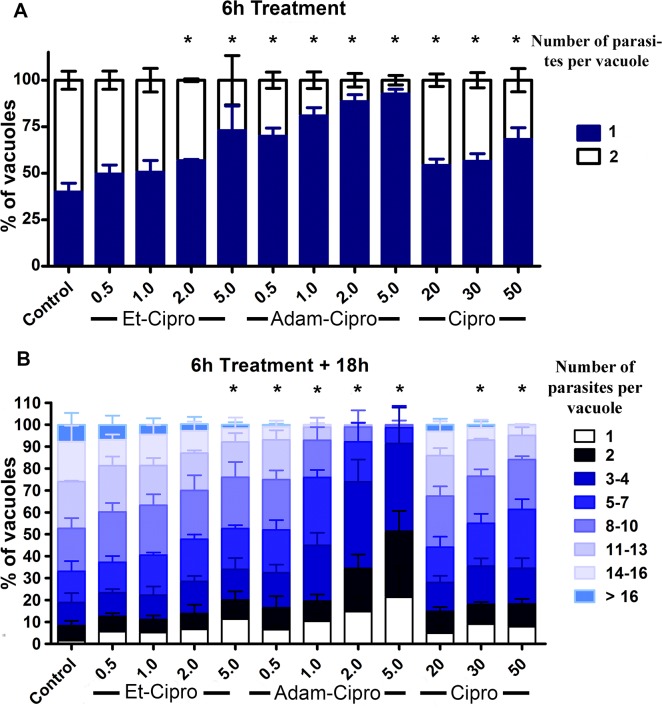
Early effects of ciprofloxacin (Cipro) derivatives in tachyzoite cell division inside host cells. (A) Tachyzoites of *T*. *gondii* were treated with Cipro derivatives for 6 h, from 1–7 h post-infection, and the number of parasites per vacuole was analyzed by light microscopy. (B) After 6 h, the medium containing drugs was removed and replaced with fresh medium without drugs. Tachyzoites were allowed to proliferate for an additional 18 h, and the number of parasites per vacuole was counted. * *P<*0.05, results were analyzed by two-way ANOVA statistical test.

Tachyzoites of the RH strain of *T*. *gondii* have a doubling time of around 6–8 h inside the parasitophorous vacuole of infected cells [25]. Treatment of infected cells with different concentrations of Et-Cipro, Adam-Cipro, and Cipro for just 6 h (7 h total infection) significantly decreased the number of parasitophorous vacuoles containing two parasites (as a result of cell division after invasion) compared with untreated controls, showing that the Cipro derivatives had an early effect on parasite division (Fig 2A). Adam-Cipro was the most active, as even low concentrations of this derivative (0.5 μM and 1 μM) significantly inhibited parasite division compared with untreated controls. The effect of treatment with 0.5 μM of Adam-Cipro was similar to that of 50 μM Cipro, with 70% and 68% of the parasitophorous vacuoles having just one parasite, respectively, corresponding to an 100-fold increase in activity for Adam-Cipro compared with the original compound. In contrast, higher concentrations of Et-Cipro (>2 μM) were required to affect parasite division significantly (Fig 2A). Nevertheless, the effect of 2 μM of Et-Cipro on early parasite division was similar to that of 20 μM of Cipro, with 57% and 54% of the vacuoles containing just one parasite, respectively, which represents a 10-fold increase in cell division inhibition compared with the original compound.

The effect of Cipro derivatives on parasite division was sustained even after these compounds were removed from the culture medium and infection was allowed to proceed for an additional 18 h (Fig 2B). The number of parasites per vacuole for all Adam-Cipro treatments was significantly lower than that observed in control, untreated cells, with less than 1% of vacuoles containing ≥16 parasites in cells treated with 0.5 μM of Adam-Cipro. After treatment with 2 or 5 μM of Adam-Cipro, approximately 99% of vacuoles contained up to eight parasites only. The presence of large numbers of vacuoles containing just one parasite 18 h after drug removal shows that some parasites were irreversibly affected by Cipro derivatives treatment.

The antiproliferative effect of Cipro derivatives and Cipro was also confirmed by plaque assay (S1 Fig). No growth was observed after 10 days treatment with 5 μM of Adam-Cipro or Et-Cipro, 10 μM of Ph-Cipro and 20 of Cipro. In contrast, untreated cultures presented many plaques. A significant decrease in parasite proliferation was also observed after treatment with 0.5 μM Adam-Cipro and 10 μM Cipro.

### Treatment with ciprofloxacin derivatives prevents daughter cell budding (but not mitosis) during tachyzoite cell division

During *T*. *gondii* cell division by ‘endodyogeny’, two daughter cells are assembled in the interior of the mother cell after a single round of DNA replication, the daughter inner membrane complex (IMC) provides ‘scaffolding’ for the newly formed and segregating daughter cell components [26, 27]. At the end of cytokinesis, the mother cell’s original IMC disassembles, and daughter cells ‘bud out’ incorporating the mother cell’s plasma membrane and leaving behind a residual body [26, 27]. Apicomplexan mitosis is ‘closed’, with the nuclear envelope remaining intact throughout the process, and being easily recognizable by its U shape [27].

**Fig 3:**
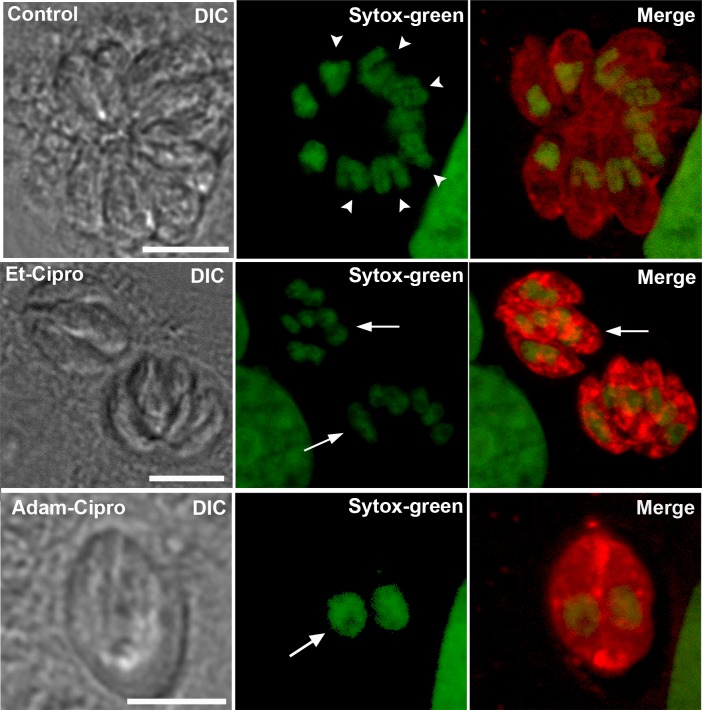
Cipro derivatives did not affect parasite mitosis. Parasites were labeled with anti-SAG1 (red, labeling the parasite’s cell surface) and Sytox green (to label the DNA). After 24 h of infection, untreated (control) parasites (top row) were typically arranged in ‘rosettes’, and displayed normal morphology, with one nucleus per cell. Many parasites were in the process of cell division, as evidenced by the presence of U-shaped mitotic nuclei (arrowheads). In cells treated with 5 μM of Et-Cipro (middle row) or Adam-Cipro (bottom row) for 24 h, tachyzoites were able to complete nuclear division, as evidenced by the presence of individualized nuclei, but could not complete cytokinesis, which led to the formation of multinucleated cells (arrows), and loss of the typical rosette organization. Images represent maximum projections of optical slices from confocal laser scanning microscopy.

**Fig 4:**
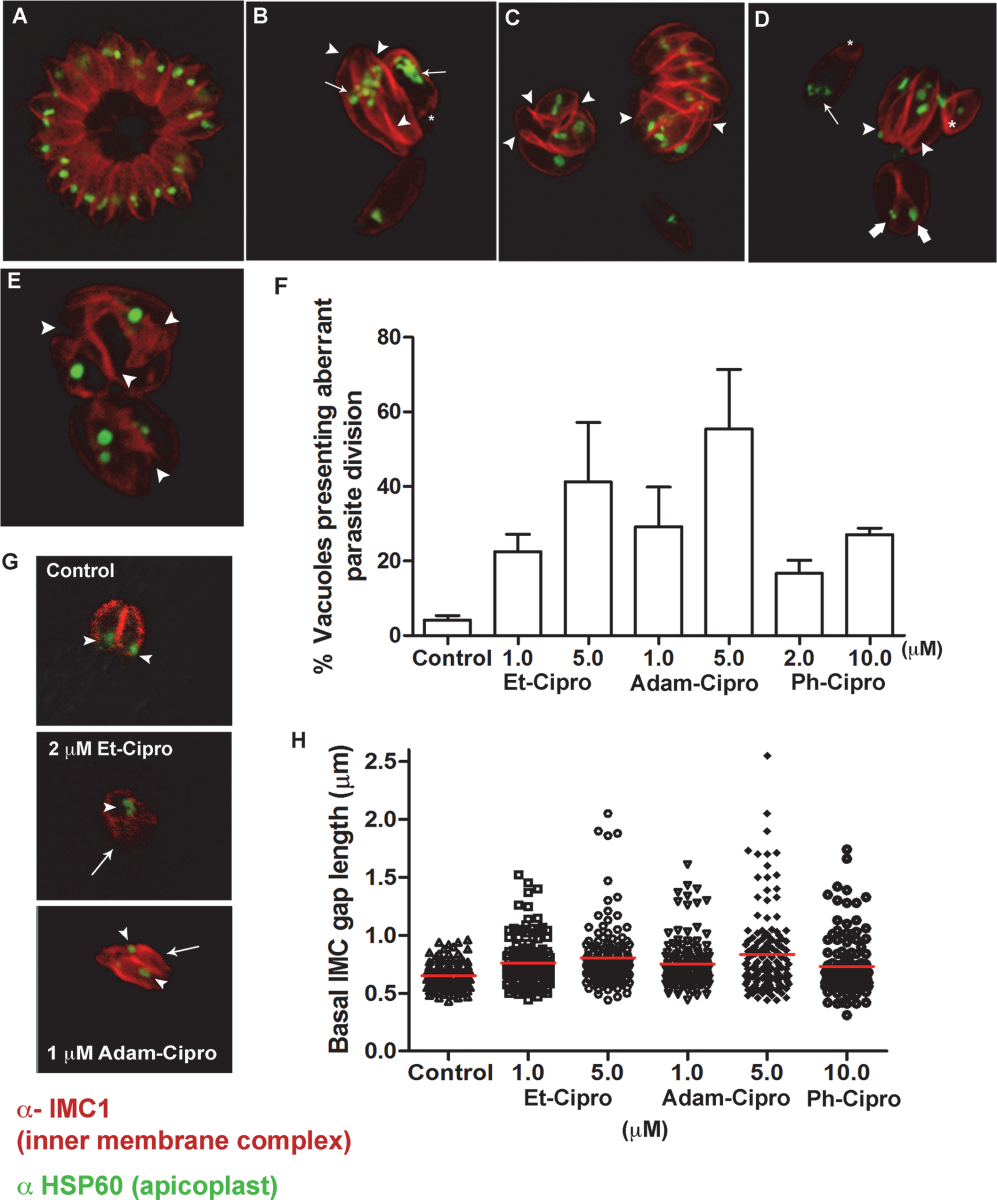
Cipro derivatives affected parasite daughter cells scission process. Immunofluorescence microscopy of LLC-MK_2_ cells infected with tachyzoites of *T*. *gondii* and treated for 24 h with Cipro derivatives. Parasites were labeled with anti-HSP60 (recognizing the apicoplast, in green) and anti-IMC1 (recognizing the inner membrane complex or IMC, in red) antibodies. (A) Untreated (control) parasites were typically organized in ‘rosettes’ and had apicoplasts of normal shape and size. Tachyzoites treated with (B) 5 μM Et-Cipro, or (C) 1 or (D) 5 μM Adam-Cipro or (E) 10 μM Ph-Cipro, for 24 h, showed enlarged and abnormally-shaped apicoplasts (arrows), signs of cell division arrest (such as ‘tethered’ parasites, arrowheads), abnormally-shaped basal complexes (asterisks), and also missegregated apicoplasts (thick arrows) that were left outside daughter cell boundaries (marked by the anti-IMC1 labelling). Images represent optical slices (untreated) or maximum projections of optical slices (Et-Cipro and Adam-Cipro). (F) Quantification of vacuoles (n = 120) containing parasites presenting cell division arrest after treatment with Cipro derivatives for 24h, results are the mean of two independent experiments. (G) Apicoplast segregation (arrowhead) and parasite division defects were observed as a result of Cipro derivative treatment in the first event of tachyzoite division inside host cells. (H) Comparison of the distribution of basal IMC gap length in untreated control (n = 143), 1 μM Et-Cipro (n = 149), 5 μM Et-Cipro (n = 144), 1 μM Adam-Cipro (n = 150), 5 μM Adam-Cipro (n = 147) and 10 μM Ph-Cipro (n = 143) after 24 h of treatment. All treatment groups had *P*<0.05 compared to control (Kruskal-Wallis statistical test and Dunn’s Multiple Comparison Test).

To elucidate the impact of treatment with Cipro derivatives on the *T*. *gondii* cell division process, we evaluated the effect of Cipro derivatives by immunofluorescence microscopy using antibody markers that recognize key parasite structures (Figs 3 and 4). Untreated parasites labeled with an antibody that recognizes SAG1, a major tachyzoite surface protein, and with Sytox-green (to label the DNA) showed a typical ‘rosette’ organization within host cells, with groups of parasites clustered with their apical/anterior ends projecting outwards, while their posterior ends clustered at the center of the rosette (untreated; Fig 3). In rosettes found in untreated cells, there was one nucleus per parasite, and some parasites were in the process of nuclear division, as evidenced by the presence of U-shaped nuclei (Fig 3, arrowheads in untreated). Treatment with 5 μM Et-Cipro and Adam-Cipro for 24 h led to the formation of large multinucleated parasites (Fig 3, arrows), with loss of the typical rosette structure.

Given that Cipro targets the apicoplast, we evaluated the effect of the new Cipro derivatives on the replication and division of this organelle, by observing dividing parasites labeled with the anti-HSP60 (Fig 4). To confirm that treatment with Cipro derivatives led to cell division arrest, cells were also labeled with an antibody that recognizes the specific IMC component IMC1 to improve visualization of daughter cell boundaries in dividing parasites (Fig 4). In non-dividing *T*. *gondii* tachyzoites, the IMC is a system of flattened vesicles associated to filamentous network found below the plasma membrane, and the combined structures of the plasma membrane and the IMC form the cell’s tri-laminar ‘pellicle’. In dividing parasites the IMC scaffolds the new daughter cells [27]. The IMC is interrupted at the micropore region, and at apical and basal ends of the parasite only [28–30]. While untreated parasites were clearly individualized and displayed only one small and round apicoplast per cell (Fig 4A), labeling with the anti-IMC1 antibody confirmed that treatment with 1 and 5 μM Et-Cipro and Adam-Cipro and 10 μM Ph-Cipro led to parasite cell division arrest, with the formation of ‘tethered’ daughter cells (Fig 4B–4E, arrowheads). Some enlarged parasites displayed IMC profiles indicative of the initiation of a new round of cell duplication prior to the completion of cytokinesis (Fig 4B–4E). Thus, the multiple tethered *T*. *gondii* cells resulted from successive rounds of cell duplication combined with cytokinesis arrest. The quantification of vacuoles presenting abnormal parasite division process showed that the division arrest by Cipro derivatives was a common effect and dose dependent (Fig 4F). Treatment with 5 μM of Adam-Cipro caused a drastic effect on tachyzoite division, leading to the arrest of endodyogeny in 55% of the parasitophorous vacuoles scored. Vacuoles containing large parasites with multiple daughter cells were also observed in parasites treated with Cipro derivatives for just 6 h and allowed to grow for additional 18 h without drugs (S2 Fig)

Parasite basal end widening (Fig 4B and 4D, asterisk) and apicoplast missegregation events (Fig 4D, large arrows) were also observed after treatment with Cipro derivatives. Additionally, some parasites treated with 5 μM Et-Cipro or Adam-Cipro displayed abnormally enlarged lobe-shaped apicoplasts (Fig 4B and 4D, arrows).

Apicoplast missegregation events were also observed by fluorescence microscopy in tachyzoites treated for just 6 h with 2 μM Et-Cipro and 1 μM Adam-Cipro (Fig 4G). In these cells, the divided apicoplast is clearly being excluded or mispositioned from one of the budding daughter cells (arrows).

The analysis of the basal IMC gap length showed that the effect of Cipro derivatives in the parasite basal end was significant after 24 h of treatment (Fig 4H). The distribution variation of the basal gap extent was higher in parasites treated with Et-Cipro, Adam-Cipro and Ph-Cipro compared to untreated control (Fig 4F). Whereas control parasites had a basal IMC length mean of 0.65 ± 0.11 μm, parasites treated with Et-Cipro, Adam-Cipro or Ph-Cipro presented a higher basal IMC length mean: 0.76 ± 0.20 μm (1 μM Et-Cipro), 0.80 ± 0.26 (5 μM Et-Cipro), 0.75 ± 0.20 (1 μM Adam-Cipro), 0.83 ± 0.34 (5 μM Adam-Cipro) and 0.73 ± 0.24 (10 μM Ph-Cipro).

### Ultrastructural analysis shows that Cipro derivatives affect IMC formation, parasite budding and apicoplast positioning

To improve our understanding of the effects of Cipro derivatives on parasite cells, the ultrastructure of treated intracellular tachyzoites was examined by TEM.

**Fig 5:**
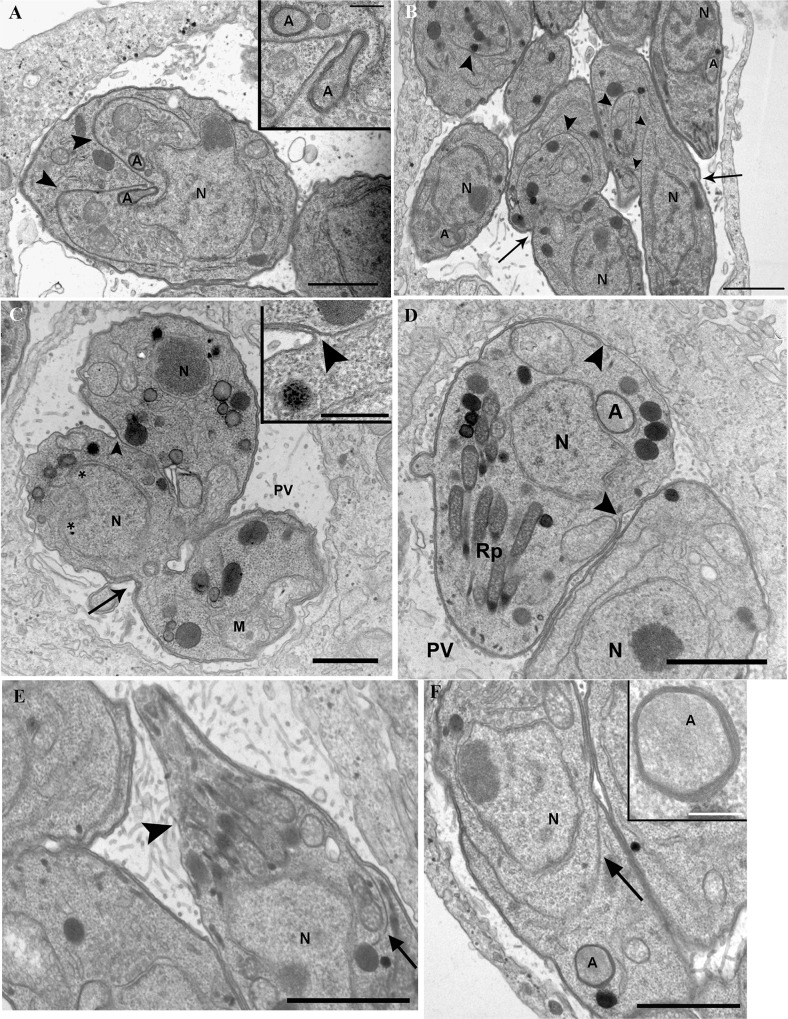
Transmission electron microscopy analysis of tachyzoites treated with Cipro derivatives. (A) Untreated cells infected with parasites undergoing normal cell division by endodyogeny. IMC (arrowheads) scaffolds daughter cells while nucleus (N) is undergoing closed mitosis. Each daughter cell is inheriting one apicoplast (inset). (B) Tachyzoites of *T*. *gondii* treated with 1 μM Et-Cipro for 48 h. Treatment led to parasite cell division arrest. Arrowheads point at daughter cells delimited by the inner membrane complex and show ‘tethered’ daughter parasites. (C) *T*. *gondii* tachyzoites treated with 5 μM Adam-Cipro for 24 h. Treatment led to parasite cell division arrest, leading to the formation of ‘tethered’ daughter cells. This derivative also affected the formation of the inner-membrane complex (IMC), and one of the daughter cells is devoid of this structure (arrowhead and inset). (D) 5 μM Et-Cipro for 24 h. Treatment increased the IMC basal gap length and apicoplast positioning. Arrowhead points to the region of the parasite’s surface devoid of an IMC (gap). (E) Tachyzoites treated with 5 μM Et-Cipro for 48 h. Part of the IMC envelope is missing (arrowhead) in the apical region of a daughter cell budding (arrow points to IMC scaffolding the daughter cell bud). (F) Tachyzoites treated with 5 μM Et-Cipro for 48 h. Treatment caused apicoplast (inset) to be positioned outside the boundaries of the daughter cell IMC (arrowhead), which is likely to lead to apicoplast missegregation during parasite division. A-apicoplast; HC-host cell; M- mitochondrion; N-nucleus; PV-parasitophorous vacuole; Rp-ropthries. Scale bars: (A) 1 μm, 0.2 μm inset; (B) 2 μm (C) 0.5 μm, 0.2 μm inset; (D) 1 μm; (E) 1 μm; (F) 1 μm, 0.2 μm inset.

In untreated control cells, observation of the parasitophorous vacuole by TEM revealed profiles of *T*. *gondii* tachyzoites undergoing a normal cell division process (Fig 5A). In untreated parasite with mitotic nuclei, newly formed IMCs (arrows) appeared to act as scaffolds for the assembling of daughter cell components, with daughter apicoplasts (insets) found in the appropriate positions for the correct segregation of one apicoplast to each daughter cell (Fig 5A).

TEM analyses of cells infected with *T*. *gondii* and treated with either 1μM Et-Cipro for 48 h (Fig 5B) or 5 μM Adam-Cipro for 24 h (Fig 5C) confirmed that these treatments led to budding arrest, with the formation of ‘tethered’ parasites. Vacuoles showing large parasites (arrows) containing multiple daughter cells (arrowheads; Fig 5B) or parasites united by their basal ends (arrow; Fig 2C) were frequently observed, suggesting incomplete cytokinesis. Treatment with Cipro derivatives also affected the IMC structure, since parasites displaying wide regions of the cell surface devoid of an underlying IMC envelope were observed after 24 h treatments with 5 μM Adam-Cipro (Fig 5C) or 5 μM Et-Cipro (arrowheads; Fig 5D–5E).

Alterations in apicoplast inheritance during parasite cell division were also evident upon TEM analysis of infected cells treated with Cipro derivatives (Fig 5D and 5F). In non-dividing tachyzoites and in forming daughter cells, the apicoplast is always found at the anterior region of the cell (Fig 5A), which is easily recognizable by the presence of a characteristic set of cytoskeletal components and secretory organelles (rhoptries and micronemes). Surprisingly, in infected cells treated with Cipro derivatives and observed by TEM, the apicoplast was often found at the posterior region of the parasite (i.e., posterior to the nucleus; Fig 5D), which is suggestive of apicoplast mispositioning during cell division and, consequently, of incorrect inheritance of this organelle by the forming daughter cells. Apicoplast missegregation was also evident in dividing tachyzoites treated with 5 μM Et-Cipro for 24 h (Fig 5F). Apicoplast is localized posterior to budding daughter cells boundaries (arrow), which implicates the loss of this organelle after completion of the division process.

### Tachyzoites treated with Cipro also shows cell division arrest, apicoplast missegregation and IMC alteration

Although the effect of Cipro in inhibiting the apicoplast genome replication has been already shown [8, 31], the phenotype induced by this drug in *T*. *gondii* has never been studied. In order to verify and confirm that the alterations caused by Cipro derivatives are specific, we also evaluated the effect of Cipro (original molecule) by immunofluorescence and TEM. For that, tachyzoites were treated with 20 μM of Cipro for 24, 48 and 72h.

**Fig 6:**
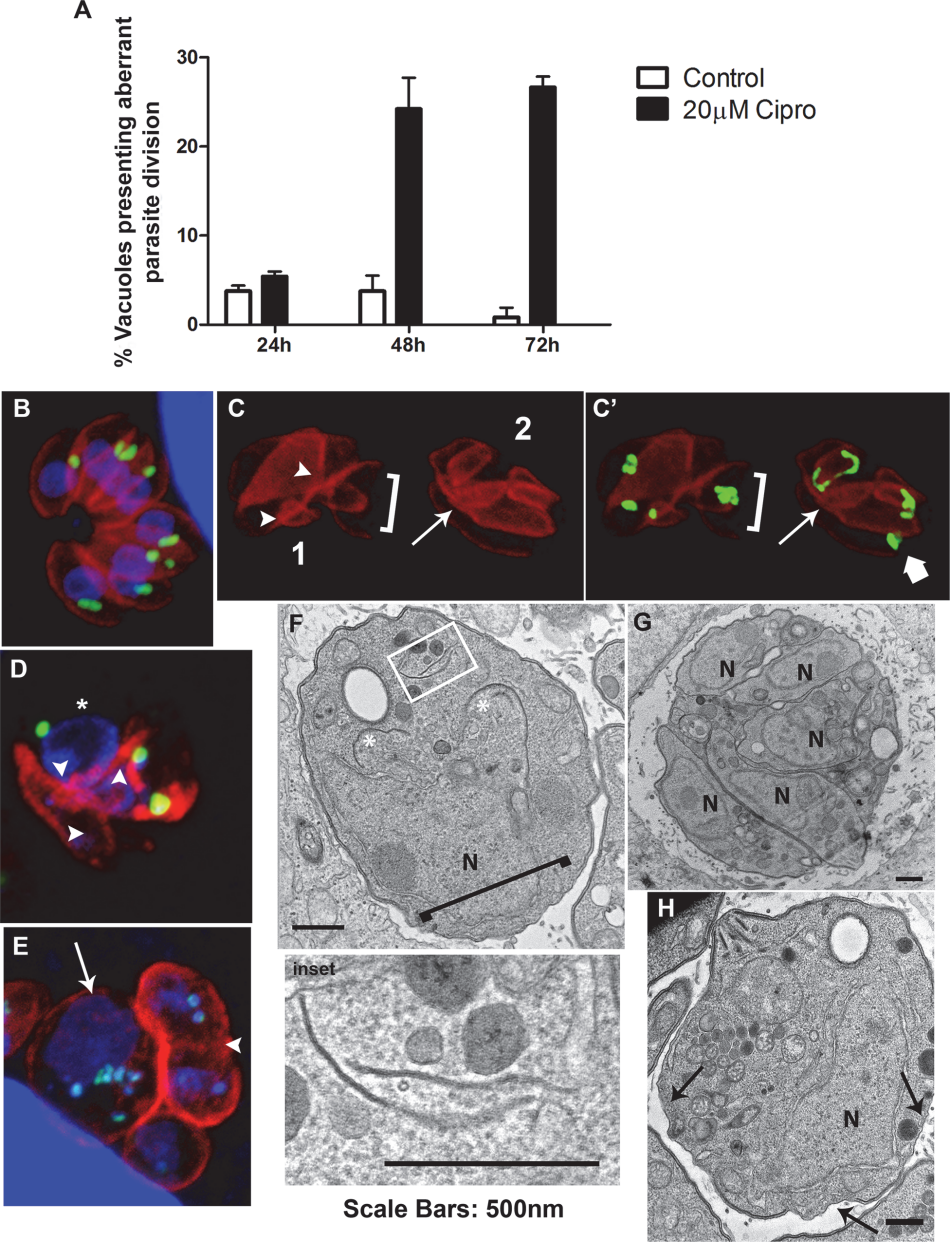
Treatment of *T*. *gondii* with Cipro also leads to cell division arrest, apicoplast missegregation and IMC alteration. Tachyzoites of *T*. *gondii* were treated with 20 μM of Cipro for 24, 48 and 72 h. (A) Quantification of vacuoles (n = 120) containing parasites presenting cell division arrest after treatment with 20 μM of Cipro for 24, 48 and 72 h, results are the mean of two independent experiments. (B-H) Immunofluorescence and TEM microscopy of LLC-MK_2_ cells infected with tachyzoites of *T*. *gondii* and treated for 48 and 72 h with 20 μM Cipro. For immunofluorescence parasites were labeled with anti-HSP60 (recognizing the apicoplast, in green), anti-IMC1 (recognizing the inner membrane complex or IMC, in red) antibodies and DAPI (to label the DNA). While untreated tachyzoites presented normal morphology (B), parasites treated for 48 and 72 h presented cell division arrest, such as ‘tethered’ parasites (C-E and G), abnormally-shaped basal complexes (C and F square brackets, and inset), missegregated (C’ arrow) and mispositioning apicoplasts (C’ thick arrows), parasites displaying regions of pellicle without IMC (asterisk in D and arrows in H) and large degenerated parasites showing abnormal nucleus size (E arrow).

While the treatment of tachyzoites with Cipro for 24h has not induced any significant alteration, the treatments for 48h and 72h caused the same phenotype observed for Cipro derivatives (Fig 6). As well as seen for Cipro derivatives, Cipro also caused cell division arrest (Fig 6A–6E). The quantification of the number of parasitophorous vacuoles containing parasites presenting cell division arrest showed that the treatment with 20 μM of Cipro for 48 and 72 h affected 24 and 26% of the vacuoles, respectively. ‘Tethered’ daughter cells were observed after 48 and 72 h of treatment with Cipro by immunofluorescence (arrowheads in Fig 6C–6E) and TEM (Fig 6G). Enlarged parasites in division process (asterisks) and containing additional IMC profiles in the cytoplasm (inset) were observed by TEM after Cipro treatment for 48 h (Fig 6F and inset). After 72 h of treatment the presence of multiple well-delimited daughter cells—the presence of a single individualized nucleus (N) by each daughter is evident—in the interior of a large parasite after (Fig 6G), points that Cipro also inhibited *T*. *gondii* the final step of cytokinesis. After Cipro treatment for 72 h, vacuoles containing large degenerated parasites showing abnormal nucleus size were also often observed (Fig 6E arrow) by immunofluorescence.

Apicoplast missegregation (arrow) and mispositioning (large arrow) events (Fig 6C and 6C’), basal end widening (square brackets in Fig 6C and 6F) and parasites displaying regions of pellicle without IMC (asterisk in Fig 6D and arrows in Fig 6H) were also observed after treatment with Cipro derivatives. In Fig 6C’ rosette 2, an elongated apicoplast was excluded of one forming daughter cell (arrow) during endodyogeny and large arrow points to an apicoplast localized at the posterior end of the parasite. Apicoplast missegregation and mispositioning during parasite division might be the cause of apicoplast loss after Cipro treatment.

### Cipro derivatives affect MORN1 localization during parasite division

Considering the crucial role of the protein MORN1 in organizing *T*. *gondii* basal complex—cytoskeletal structure at the basal end of the parasite [32]—and to promote daughter cell scission in the division process [33–35], we evaluated the distribution of MORN1 after treatment with Cipro derivatives and Cipro by immunofluorescence using a specific antibody against this protein.

**Fig 7:**
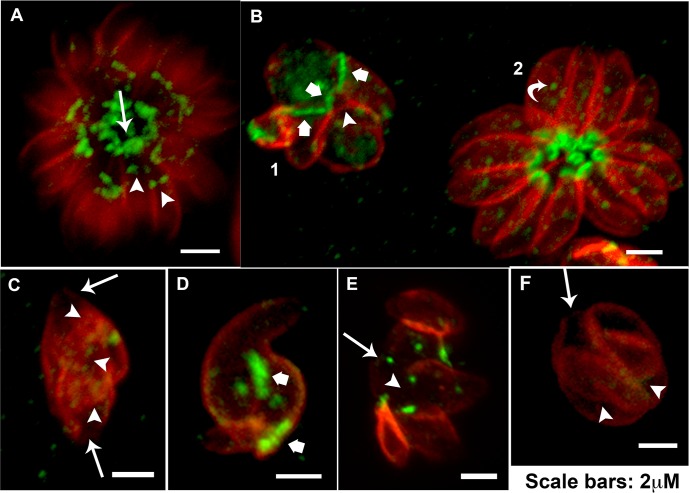
Cipro derivatives and Cipro affect MORN1 localization during parasite division. In parasites presenting normal division process MORN1 localizes at the basal complex of the mother cell (arrow) and also caps the final end of the IMC in budding daughter cells (arrowheads) (A), a single strong point in the middle of the cell was also observed in normal non-dividing parasites (B rosette 2 curved arrow). Tethered daughter cells resulting from the treatment with 5 μM Et-Cipro (B-C) and Adam-Cipro (D-E) for 24h, and 20 μM Cipro for 72h (F) showed wide MORN1 basal caps (B and D large arrows) and lack or a weak deposition of MORN1, at the basal end of mother (arrow) and budding daughter cells (arrowheads) (B-F)

MORN1 localizes at the basal complex of the parasite (arrow) and also at the final end of the IMC in budding daughter cells during division (arrowheads) (Fig 7A). A point in the middle of the cell, corresponding to the centrocone (a cone shape structure in the nuclear envelope that houses the mitotic spindle [26] was also observed (Fig 7B, curved arrow in rosette 2).

Treatment with 5 μM of Et-Cipro or Adam-Cipro for 24 h and also 20 μM of Cipro for 72h led to a drastic effect in MORN1 distribution and localization, mainly in dividing parasites (Fig 7B–7F). Tethered daughter cells presenting a wide basal complex (about 2 μm extension), evidenced by MORN1 cap, were observed after treatment with 5 μM of Et-Cipro or Adam-Cipro (Fig 7B and 7D, large arrows). However, tethered daughter cells often presented a weak localization or lacked MORN1 at the end regions of IMC in mother cell (arrows) and also in budding daughter cells (arrowheads) after the treatment with 5 μM of Et-Cipro or Adam-Cipro for 24 h and 20 μM of Cipro for 72 h (Fig 7B, 7C, 7E and 7F). Taking into account the importance of MORN1 for the assembly of basal complex, and daughter cells scission [35], the lack of this protein at this region could be associated to the inhibition of daughter cells scission after Cipro and Cipro derivatives treatment.

### Cipro derivatives are less active against the apicomplexan *C*. *parvum*

The Apicomplexa species *C*. *parvum* lacks an apicoplast [36]. Thus, we hypothesized that if Cipro derivatives target the apicoplast, these compounds would be less effective at inhibiting *C*. *parvum* proliferation,. To test this hypothesis, the biological activities of Cipro derivatives were determined by measuring the inhibition of *C*. *parvum* growth in MDBK cells (Table 2). Treatments with 50 μM of Ph-Cipro and Adam-Cipro were toxic to host cells (data not shown) and 50 μM of Et-Cipro just inhibited 36% of *C*. *parvum* proliferation; despite the toxicity for the host cells, the treatment with 50 μM of Ph-Cipro and Adam-Cipro did not show to decrease the proliferation of *C*. *parvum*, as infected cells showed a number of parasites similar to the cultures treated with 25 μM (data not shown). Therefore, the IC50 value was only estimated for Cipro (39.2 μM after 24h; Table 2).

**Table 2:** Effect of treatment with Cipro, Et-Cipro, Adam-Cipro and Ph-Cipro on the growth of *Cryptosporidium parvum* sporozoites in host cells.

Compound	% inhibition of sporozoites growth^a^		
	12.5 μM	25 μM	50 μM
Cipro	19.5 ± 1.1	46.1 ± 2.9	53.40 ± 2.51
Et-Cipro	20.3 ± 10.1	32.6 ± 7.6	36.43 ± 9.76
Adam-Cipro	24.7 ± 8.4	34.2 ± 3.8	-
Ph-Cipro	33.6 ± 11.6	42.6 ± 14.6	-

^a^ The % sporozoite growth inhibition was calculated by the examination of at least 1000 MDBK cells.

## Discussion

Few therapy options are currently available for toxoplasmosis treatment, and effective drugs have serious toxic effects, leading to treatment interruption. The discovery of the apicoplast in the phylum Apicomplexa – which includes *T*. *gondii* and other important human parasites, such as *Plasmodium* spp.—brought new perspectives for the development of chemotherapy against these parasites, represented by the targeting of the multiple metabolic routes of prokaryotic origin found in this organelle, and which differ significantly from those found in mammalian cells. Inhibitors of apicoplast enzymes, such as azithromycin and clindamycin (which block prokaryotic protein synthesis), are already used in the treatment of human infections caused by apicomplexan parasites [5, 37]. In a previous study, we showed that derivatives of the antibiotic ciprofloxacin, a fluoroquinolone inhibitor of prokaryotic type II topoisomerases (DNA gyrase and topoisomerase IV), are active against *T*. *gondii* tachyzoites *in vitro* [20]. Here, we show that Cipro derivatives also increase the survival of mice infected with *T*. *gondii*, and affect parasite cell division by inhibiting cytokinesis and disturbing apicoplast duplication and segregation.

The *in vivo* studies showed that Cipro derivative administration was well tolerated (Table 1) and did not result in serious toxicity to mice, and only mild toxicity was observed after treatment with high doses (200mg/kg) of Ph-Cipro (Table 1). While all mice treated with Cipro died by day 10 post-infection, some mice treated with Cipro derivatives remained alive for at least 60 days, suggesting that Cipro derivatives cured *T*. *gondii* infection in treated mice. Considering that we used an acute model of infection, the modest rate of mice survival (13–25%) after treatment with Cipro derivatives is not irrelevant. High mice mortality was also observed in acute model of infection after the administration of drugs used for the treatment of toxoplasmosis in the clinic [38–40]. Thus, Cipro derivatives should be considered potentially promising for the treatment of toxoplasmosis.

Fichera and Ross (1997) [8] showed that treatment of *T*. *gondii* with Cipro led to progressive loss of apicoplast DNA, without affecting the first round of parasite proliferation in the host cells. However, egressed parasites that had been treated with Cipro suffered a drastic and permanent reduction in the rate of cell duplication in the second round of proliferation in a new host cell [8]. This ‘delayed effect’ of drugs on parasite proliferation was also seen after treatment of *T*. *gondii* and *P*. *falciparum* with clindamycin [41, 42].

Interestingly, our results show that esterified Cipro derivatives did not trigger the same delayed pattern of parasite death observed after treatment of infected cells with Cipro [8]. Rather, Et-Cipro and Adam-Cipro inhibited the first cell division event within the first round of parasite proliferation in host cells (Fig 2), since treatment with these Cipro derivatives for just 6 h led to a dramatic reduction in the number of parasitophorous vacuoles containing 2 parasites, 7h post-infection (Fig 2A). A possible explanation for these results is that esterified compounds act as prodrugs and this change would make them more lipophilic, favoring the diffusion across biological membranes (mainly the multiple membranes of *T*. *gondii* and of the apicoplast) and thus would affect apicoplast in a faster manner than the original molecule (Cipro). It is noteworthy to mention that Et-Cipro is a mere Cipro precursor, i.e. once inside the cell, Et-Cipro ethyl ester group is hydrolysed by the action of intracellular esterases and then this derivative is converted to Cipro (original molecule). Thus, the antiproliferative effect and the morphological alterations caused by Et-Cipro derivative against *T*. *gondii* can be credited to Cipro itself. An immediate effect on the proliferation of *T*. *gondii* was also observed after treatment with the fluoroquinolones gatifloxacin and trovafloxacin [43,44]. So that more active compounds seems to subvert the delayed effect on apicoplast. The IC_50_ values obtained for gatifloxacin (0.52 μM in HFF primary cell culture after 48h) and trovafloxacin (1.37 μM in L929 cell line) against *T*. *gondii* [43,44] were close to those obtained for Cipro derivatives (0.42 μM Et-Cipro, 0.46 μM Adam-Cipro and 1.24 μM Ph-Cipro after 48h) [20]. The increased activity by ethyl-esterified Cipro derivatives had also been previously reported in *P*. *falciparum* [45]. Indeed, Cipro had an immediate effect against *P*. *falciparum* and did not show the delayed phenotype [46]. Therefore, the delayed effect of Cipro against *T*. *gondii* is possibly due to its poor diffusion through *T*. *gondii* membranes.

Although Cipro shows a delayed effect against *T*. *gondii*, treatment with this drug induced a set of alterations identical to those caused by Cipro derivatives treatments (Figs 4–7) and points that they are acting in the same target. Moreover, the biological activity of Cipro derivatives against *C*. *parvum*, which lacks an apicoplast (Table 2), is, approximately, 50 fold lower than that in *T*. *gondii* tachyzoites [20], reinforcing the notion that treatment with Cipro derivatives affects the apicoplast directly. The effect of Cipro against this apicomplexa, however, is not surprising, as a moderate effect of Cipro against *C*. *parvum* has been reported before [47], and Cipro is possibly acting by an off target in this parasite.

Homologous genes for subunit A of the DNA gyrase—the putative target of the Cipro derivatives used in this study—were identified in the nuclear genome of apicomplexans, and their protein products are targeted to the apicoplast [18,19]. Thus, it is not surprising that we observed apicoplast alteration after treatment with Cipro derivatives and Cipro (Figs 4B–4D, 5D, 5F and 6C’). Our immunofluorescence and TEM data strongly suggest that the apicoplast loss observed in some cells after Cipro derivatives and Cipro treatments is likely caused by apicoplast missegregation during cell division (Figs 4D–4E, 5D, 5F and 6C’). Apicoplast division is concomitant with mitosis and is closely associated with the centrosome and with the nuclear spindle poles [48, 49], which is likely to ensure the correct segregation of this organelle to daughter cells [48]. To date, no protein involved in the physical link between the apicoplast and the centrosome has been identified.

Immunofluorescence and TEM analyses suggest that, aside from apicoplast duplication and segregation inhibition, treatment with Cipro derivatives also affected the final step of parasite cytokinesis (cell scission), which involves constriction at the basal end of dividing parasites and cell separation. These observations suggest that parasites were able to perform mitosis (Fig 3), but were unable to complete cell scission at the end of cytokinesis, which occurs just after daughter cells bud out of the mother cell [27]. Consequently, parasites remained linked together at their basal ends, leading to the formation of groups of tethered parasites. This might have been the main effect of Cipro derivative treatment, since it was also visualized in parasites treated with a concentration close to the IC_50_ (1 μM Et-Cipro or Adam-Cipro; Fig 4C and 4F). Cell scission inhibition was also observed after treatment with 20 μM Cipro for 48 and 72h (Fig 6A–6E). Treatment with Cipro also led to impairment of *P*. *falciparum* cell division. After treatment with Cipro, parasites were able to initiate cell division, but failed in forming mature schizonts [50].

The immediate cell scission inhibition by Cipro derivatives is likely responsible for the strong parasite proliferation inhibition caused by Et-Cipro and Adam-Cipro treatments, which led to the formation of ‘tethered’ parasites, where multiple rounds of cell duplication had occurred in the absence of cytokinesis completion. The presence of vacuoles containing multiple tethered daughter cells even after Cipro derivatives (Et-Cipro and Adam-Cipro) removal (S2 Fig) showed that cell scission inhibition was irreversible, as daughter cells forming from new division cycles after drug removal were also unable to complete cytokinesis.

Our TEM and immunofluorescence results suggest that Cipro derivatives and Cipro treatments affected the integrity of IMC in mother cell (Fig 6H), as well as, the formation of this structure in daughter cells during cell division (Figs 5C, 5F, and 6D). The IMC supports the subpellicular microtubules [29], is important for parasite motility and host cell invasion [51], and serves as a scaffold for newly assembled structures during daughter cell formation [27, 52]. During daughter cell budding, daughter IMCs is assembled *de novo* [53], which possibly requires large amounts of lipids. Although *T*. *gondii* can obtain fatty acids from the host cell [54], apicoplast fatty acid synthesis is also essential for parasite survival [11, 55], since the apicoplast is likely the main source of short-chain fatty acids (C14:0 and C16:0), and cooperates with the endoplasmic reticulum in the synthesis of long-chain fatty acid [55]. Thus, loss of apicoplast function by Cipro derivative treatment is expected to affect fatty acid synthesis and IMC formation.

Other intriguing finding is the lack of MORN1 deposition in the basal end of tethered daughter cells in parasites presenting cell division inhibition after treatment with Cipro derivatives and Cipro (Fig 7). MORN1 is the major basal complex component and ‘caps’ the posterior (or basal) end of the parasite [48, 49]. At the very beginning of daughter buds assembling, Morn1 forms a pair of rings around early duplicated centrioles [32, 33], and caps and migrates in synchrony with the IMC during daughter cells budding [33, 56]. Late in cytokinesis, the basal complex protein components constrict the basal end of newly formed parasites, eventually ‘resolving’ cell division [32–34, 56]. MORN1 showed an essential role in the assembly and maturation of basal complex, in its absence other components of the basal complex such as, Centrin2, dynein light chain and a set of basal IMC proteins could not assemble in the basal end of the parasite [32–35]. Knockout of MORN1 leads to cytokinesis and apicoplast segregation defects [34, 35], with the formation of ‘Janus-headed’ parasites [34] and also parasites with basal end wider than normal [35]. This phenotype is similar to the effect of Cipro derivatives and Cipro treatments described here (Figs 4B–4G, 5B, 5C and 6A–6G). By immunofluorescence microscopy, we observed tethered tachyzoites with a wide basal end after Cipro derivative treatment (Figs 4B, 4D, 6C and 7B and 7D), and TEM images of parasites treated with 5 μM Et-Cipro and 20 μM Cipro show tachyzoites with a wider basal region devoid of an underlying IMC envelope (arrowheads in Fig 5D and square bracket in Fig 6F, respectively). Thus, the inability of parasites treated with Cipro derivatives and Cipro to perform cell scission might be due to impaired basal complex maturation due to the lack of MORN1.

Overall, our *in vivo* and *in vitro* studies show that Cipro derivatives deserve to be exploited further in the search for alternative chemotherapeutic agents to treat human toxoplasmosis.

## Supporting Information

S1 Fig Plaque assay showing the antiproliferative effect of Cipro derivatives after 10 days treatment. While untreated culture (control) shows diverse plaques, treatment with just 0.5 μM of Adam-Cipro or 10 μM Cipro led to a drastic reduction on parasite proliferation. Indeed, no plaques were observed in cultures treated with 5 μM of Adam-Cipro or Et-Cipro, 10 μM of Ph-Cipro and 20 μM of Cipro.
(TIF)

S2 Fig The effect of Cipro derivatives in *T*. *gondii* division is irreversible. Immunoluorescence microscopy of tachyzoites labeled with anti-HSP60 (recognizing the apicoplast, in green) and anti-IMC1 (recognizing the inner membrane complex or IMC, in red) antibodies, and treated with Cipro derivatives for 6 h and then observed and kept in culture for a further 18h in the absence of drugs. Parasite division defects were observed (arrows) as a result of Cipro derivative treatment in the first event of tachyzoite division inside host cells. Even after drug removal from the medium, cell division continued to be affected. Images represent maximum projection of optical slices.
(TIF)

## References

[1] McLeod R, Kieffer F, Sautter M, Hosten T, Pelloux H. Why prevent, diagnose and treat congenital toxoplasmosis? Mem Inst Oswaldo Cruz. 2009, 104: 320–44. 19430661 10.1590/s0074-02762009000200029 PMC2735102

[2] Pereira-Chioccola VL, Vidal JE, Su C. Toxoplasma gondii infection and cerebral toxoplasmosis in HIV-infected patients. Future Microbiol. 2009, 4:1363–79. 10.2217/fmb.09.89 19995194

[3] Arevalo JF, Belfort R Jr, Muccioli C, Espinoza JV. Ocular toxoplasmosis in the developing world. Int Ophthalmol Clin. 2010, 50:57–69. 10.1097/IIO.0b013e3181f0faee 20375862

[4] Holland GN. Ocular toxoplasmosis: a global reassessment. Part I: epidemiology and course of disease. Am J Ophthalmol. 2003, 136: 973–988. 14644206 10.1016/j.ajo.2003.09.040

[5] Montoya JG, Liesenfeld O. Toxoplasmosis. Lancet. 2004, 63:1965–76. 10.1016/S0140-6736(04)16412-X 15194258

[6] Iaccheri B, Fiore T, Papadaki T, Androudi S, Janjua S, Bhaila I, et al Adverse drug reactions to treatments for ocular toxoplasmosis: a retrospective chart review. Clin Ther. 2008, 30:2069–74. 10.1016/j.clinthera.2008.10.021 19108794

[7] Köhler S, Delwiche CF, Denny PW, Tilney LG, Webster P, Wilson RJ, et al A plastid of probable green algal origin in Apicomplexan parasites. Science. 1997, 275:1485–9. 9045615 10.1126/science.275.5305.1485

[8] Fichera M, Roos DS. A plastid organelle as a drug target in apicomplexan parasites. Nature. 1997, 390: 407–9. 9389481 10.1038/37132

[9] Goodman CD, McFadden GI. Targeting apicoplasts in malaria parasites. Expert Opin Ther Targets. 2013, 17: 167–77. 10.1517/14728222.2013.739158 23231425

[10] van Dooren GG, Striepen B. The algal past and parasite present of the apicoplast. Annu Rev Microbiol. 2013, 67: 271–89. 10.1146/annurev-micro-092412-155741 23808340

[11] Mazumdar J, Wilson EH, Masek K, Hunter CA, Striepen B. Apicoplast fatty acid synthesis is essential for organelle biogenesis and parasite survival in Toxoplasma gondii. Proc Natl Acad Sci U S A. 2006, 103: 13192–7. 16920791 10.1073/pnas.0603391103 PMC1559775

[12] McFadden GI, Waller RF. Plastids in parasites of humans. Bioassays. 1997, 19: 1033–40. 9394626 10.1002/bies.950191114

[13] Wiesner J, Reichenberg A, Heinrich S, Schlitzer M, Jomaa H. The plastid-like organelle of apicomplexan parasites as drug target. Curr Pharm Des. 2008, 14:855–71. 18473835 10.2174/138161208784041105

[14] Collin F, Karkare S, Maxwell A. Exploiting bacterial DNA gyrase as a drug target: current state and perspectives. Appl Microbiol Biotechnol. 2011, 92: 479–97. 10.1007/s00253-011-3557-z 21904817 PMC3189412

[15] Gouvea LR, Martins DA, Batista Dda G, Soeiro Mde N, Louro SR, Barbeira PJ, et al Norfloxacin Zn(II)-based complexes: acid base ionization constant determination, DNA and albumin binding properties and the biological effect against *Trypanosoma cruzi* . Biometals. 2013, 26: 813–25. 10.1007/s10534-013-9661-z 23897315

[16] Hooper D. Mechanisms of action of antimicrobials: focus on fluoroquinolones. Clin Infect Dis. 2001, 32: S9–S15. 11249823 10.1086/319370

[17] Johannes CB, Ziyadeh N, Seeger JD, Tucker E, Reiter C, Faich G. Incidence of allergic reactions associated with antibacterial use in a large, managed care organisation. Drug Saf. 2007, 30: 705–13. 17696583 10.2165/00002018-200730080-00007

[18] Dar MA, Sharma A, Mondal N, Dhar SK. Molecular cloning of apicoplast-targeted Plasmodium falciparum DNA gyrase genes: unique intrinsic ATPase activity and ATP-independent dimerization of PfGyrB subunit. Eukaryot Cell. 2007, 6: 398–412. 17220464 10.1128/EC.00357-06 PMC1828931

[19] Raghu Ram EV, Kumar A, Biswas S, Kumar A, Chaubey S, et al Nuclear gyrB encodes a functional subunit of the *Plasmodium falciparum* gyrase that is involved in apicoplast DNA replication. Mol Biochem Parasitol. 2007, 154: 30–39. 17499371 10.1016/j.molbiopara.2007.04.001

[20] Dubar F, Wintjens R, Martins-Duarte ES et al Ester prodrugs of Ciprofloxacin as DNA-gyrase inhibitors: synthesis, antiparasitic evaluation and docking studies. Med Chem Commun. 2011, 2: 430–5.

[21] Martins-Duarte EdosS, de Souza W, Vommaro RC. Itraconazole affects *Toxoplasma gondii* endodyogeny. FEMS Microbiol Lett. 2008, 282: 290–8. 10.1111/j.1574-6968.2008.01130.x 18371067

[22] da Silva CF, Batista Dda G, Oliveira GM, de Souza EM, Hammer ER, et al In vitro and in vivo investigation of the efficacy of arylimidamide DB1831 and its mesylated salt form—DB1965—against Trypanosoma cruzi infection. PLoS One. 2012, 7:e30356 10.1371/journal.pone.0030356 22291940 PMC3264605

[23] Lawton P, Naciri M, Mancassola R, Petavy AF. In vitro cultivation of Cryptosporidium parvum in the non-adherent human monocytic THP-1 cell line. J Eukaryot Microbiol. 1997, 44: 66S 9508448 10.1111/j.1550-7408.1997.tb05783.x

[24] Lawton P, Hejl C, Mancassola R, Naciri M, Petavy AF. Effects of purine nucleosides on the in vitro growth of Cryptosporidium parvum. FEMS Microbiol Lett. 2003, 226:39–43. 13129605 10.1016/S0378-1097(03)00555-X

[25] Roos DS, Donald RG, Morrissette NS et al Molecular tools for genetic dissection of the protozoan parasite *Toxoplasma gondii* . Methods Cell Biol. 1994, 45:27–63. 7707991 10.1016/s0091-679x(08)61845-2

[26] Sheffield HG, Melton ML. The fine structure and reproduction of *Toxoplasma gondii* . J Parasitol. 1968, 54: 209–26. 5647101

[27] Hu K, Mann T, Striepen B, Beckers CJ, Roos DS, et al (2002) Daughter cell assembly in the protozoan parasite Toxoplasma gondii. Mol Biol Cell 13: 593–606. 11854415 10.1091/mbc.01-06-0309 PMC65652

[28] Nichols BA, Chiappino ML. Cytoskeleton of Toxoplasma gondii. J Protozool. 1987, 34:217–26. 3585817 10.1111/j.1550-7408.1987.tb03162.x

[29] Morrissette NS, Murray JM, Roos DS. Subpellicular microtubules associate with an intramembranous particle lattice in the protozoan parasite *Toxoplasma gondii* . J Cell Sci. 1997, 110: 35–42. 9010782 10.1242/jcs.110.1.35

[30] Mann T, Beckers C. Characterization of the subpellicular network, a filamentous membrane skeletal component in the parasite Toxoplasma gondii. Mol Biochem Parasitol. 2001, 115:257–68. 11420112 10.1016/s0166-6851(01)00289-4

[31] Weissig V, VetroWidenhouse TS, Rowe TC. Topoisomerase II inhibitors induce cleavage of nuclear and 35-kb plastid DNAs in the malarial parasite Plasmodium falciparum. DNA Cell Biol. 1997, 16: 1483–92. 9428797 10.1089/dna.1997.16.1483

[32] Hu K. Organizational changes of the daughter basal complex during the parasite replication of Toxoplasma gondii. PLoS Pathog. 2008, 4:e10 10.1371/journal.ppat.0040010 18208326 PMC2211554

[33] Gubbels MJ, Vaishnava S, Boot N, Dubremetz JF, Striepen B. A MORN-repeat protein is a dynamic component of the Toxoplasma gondii cell division apparatus. J Cell Sci. 2006, 119: 2236–45. 16684814 10.1242/jcs.02949

[34] Lorestani A, Sheiner L, Yang K, Robertson SD, Sahoo N, et al A Toxoplasma MORN1 null mutant undergoes repeated divisions but is defective in basal assembly, apicoplast division and cytokinesis. PLoS One. 2010, 5: e12302 10.1371/journal.pone.0012302 20808817 PMC2924399

[35] Heaslip AT, Dzierszinski F, Stein B, Hu K. TgMORN1 is a key organizer for the basal complex of Toxoplasma gondii. PLoS Pathog. 2010, 6:e1000754 10.1371/journal.ppat.1000754 20140195 PMC2816694

[36] Zhu G, Marchewka MJ, Keithly JS. *Cryptosporidium parvum* appears to lack a plastid genome. Microbiology. 2000, 146:315–321. 10708370 10.1099/00221287-146-2-315

[37] Fleige T, Soldati-Favre D. Targeting the transcriptional and translational machinery of the endosymbiotic organelle in apicomplexans. Curr Drug Targets. 2008, 9:948–56. 18991607 10.2174/138945008786786073

[38] Martins-Duarte ES, de Souza W, Vommaro RC. Toxoplasma gondii: the effect of fluconazole combined with sulfadiazine and pyrimethamine against acute toxoplasmosis in murine model. Exp Parasitol. 2013, 133: 294–9. 10.1016/j.exppara.2012.12.011 23270807

[39] Romand S, Pudney M, Derouin F.) In vitro and in vivo activities of the hydroxynaphtoquinone atovaquone alone or combined with pyrimethamine, sulfadiazine, clarithromycin, or minocycline against Toxoplasma gondii. Antimicrob Agents Chemother. 1993, 37: 2371–8. 8285620 10.1128/aac.37.11.2371 PMC192394

[40] Derouin F, Almadany R, Chau F, Rouveix B, Pocidalo JJ. Synergistic activity of azithromycin and pyrimethamine or sulfadiazine in acute experimental toxoplasmosis. Antimicrob Agents Chemother. 1992, 36: 997–1001. 1324642 10.1128/aac.36.5.997 PMC188824

[41] Fichera ME, Bhopale MK, Roos DS. In vitro assays elucidate peculiar kinetics of clindamycin action against Toxoplasma gondii. Antimicrob Agents Chemother. 1995, 39: 1530–7. 7492099 10.1128/aac.39.7.1530 PMC162776

[42] Pfefferkorn ER, Borotz SE. Comparison of mutants of Toxoplasmagondii selected for resistance to azithromycin, spiramycin, or clindamycin.Antimicrob. Agents Chemother. 1994, 38:31–37. 8141576 10.1128/aac.38.1.31 PMC284392

[43] Khan A, Slifer T, Araujo F, Remington JS. Trovafloxacin is active against *Toxoplasma gondii* . Antimicrob Agents Chemother. 1996, 40: 1855–9. 8843293 10.1128/aac.40.8.1855 PMC163429

[44] Khan A, Slifer T, Araujo F, Remington JS. Activity of gatifloxacin alone or in combination with pyrimethamine or gamma interferon against *Toxoplasma gondii* . Antimicrob Agents Chemother. 2001, 45: 48–51. 11120943 10.1128/AAC.45.1.48-51.2001 PMC90238

[45] Dubar F, Anquetin G, Pradines B et al Enhancement of the antimalarial activity of Ciprofloxacin using a double prodrug/bioorganometallic approach. J Med Chem. 2009, 24: 7954–7. 10.1021/jm901357n 19908867

[46] Goodman CD, Su V, McFadden GI. The effects of anti-bacterials on the malaria parasite Plasmodium falciparum. Mol Biochem Parasitol. 2007, 152: 181–191. 17289168 10.1016/j.molbiopara.2007.01.005

[47] Woods KM, Nesterenko MV, Upton SJ. Efficacy of 101 antimicrobials and other agents on the development of Cryptosporidium parvum in vitro. Ann Trop Med Parasitol. 1996, 90; 603–615 9039272 10.1080/00034983.1996.11813090

[48] Striepen B, Crawford MJ, Shaw MK, Tilney LG, Seeber F, et al The plastid of toxoplasma gondii is divided by association with the centrosomes. J Cell Biol. 2000, 151: 1423–34. 11134072 10.1083/jcb.151.7.1423 PMC2150670

[49] Nishi M, Hu K, Murray JM, Roos DS. Organellar dynamics during the cell cycle of *Toxoplasma gondii* . J Cell Sci. 2008, 121:1559–68. 10.1242/jcs.021089 18411248 PMC6810632

[50] Dahl EL, Rosenthal PJ. Multiple antibiotics exert effects against the Plasmodium falciparum apicoplast. Antimicrob Agents Chemother. 2007, 51:3485–90. 17698630 10.1128/AAC.00527-07 PMC2043295

[51] Gaskins E, Gilk S, DeVore N et al Identification of the membrane receptor of a class XIV myosin in *Toxoplasma gondii* . J Cell Biol. 2004; 165: 383–93. 15123738 10.1083/jcb.200311137 PMC2172186

[52] Beck JR, Rodriguez-Fernandez IA, de Leon JC, Huynh MH, Carruthers VB, Morrissette NS, et al A novel family of Toxoplasma IMC proteins displays a hierarchical organization and functions in coordinating parasite division. PLoS Pathog. 2010, 6:e1001094. 20844581 10.1371/journal.ppat.1001094 PMC2936552

[53] Ouologuem DT, Roos DS. Dynamics of the Toxoplasma gondii inner membrane complex. J Cell Sci. 2014, 127:3320–30. 10.1242/jcs.147736 24928899 PMC4134349

[54] Charron AJ, Sibley LD. Host cells: mobilizable lipid resources for the intracellular parasite *Toxoplasma gondii* . J Cell Sci. 2002, 115:3049–59. 12118061 10.1242/jcs.115.15.3049

[55] Ramakrishnan S, Docampo MD, Macrae JI, Pujol FM, Brooks CF, van Dooren GG, et al Apicoplast and endoplasmic reticulum cooperate in fatty acid biosynthesis in apicomplexan parasite Toxoplasma gondii. J Biol Chem. 2012, 287:4957–71. 10.1074/jbc.M111.310144 22179608 PMC3281623

[56] Hu K, Johnson J, Florens L, Fraunholz M, Suravajjala S, DiLullo C, et al Cytoskeletal components of an invasion machine—the apical complex of Toxoplasma gondii. PLoS Pathog. 2006, 2:e13 16518471 10.1371/journal.ppat.0020013 PMC1383488

