# Supplementary figures and images for: Ciprofloxacin Derivatives Affect Parasite Cell Division and Increase the Survival of Mice Infected with Toxoplasma gondii
Source: PLoS One. 2015 May 7;10(5):e0125705. doi: 10.1371/journal.pone.0125705 (PMC4423777; doi:10.1371/journal.pone.0125705)

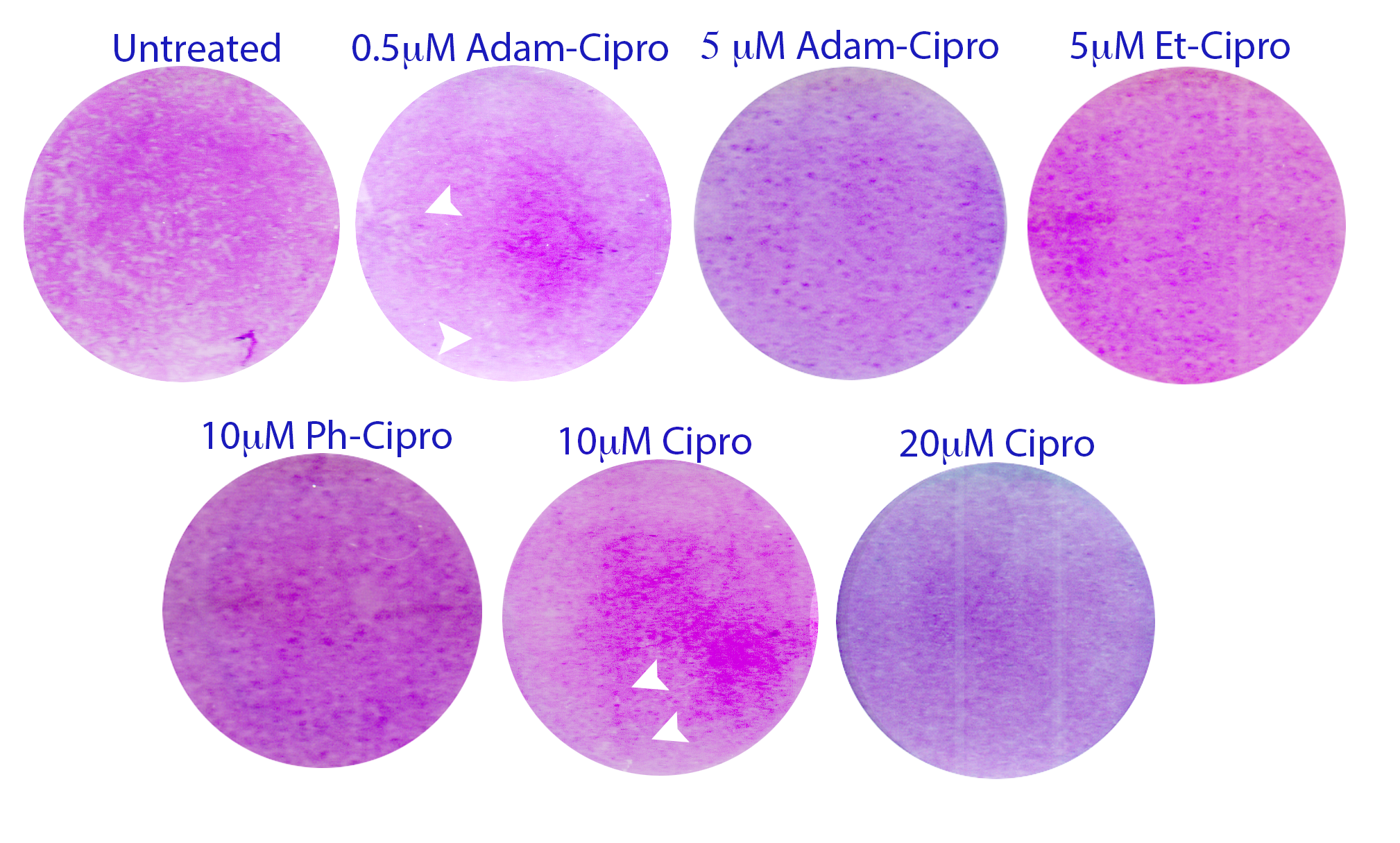

Supplement: S1 Fig — While untreated culture (control) shows diverse plaques, treatment with just 0.5 μM of Adam-Cipro or 10 μM Cipro led to a drastic reduction on parasite proliferation. Indeed, no plaques were observed in cultures treated with 5 μM of Adam-Cipro or Et-Cipro, 10 μM of Ph-Cipro and 20 μM of Cipro. (TIF) [file pone.0125705.s001.tif]

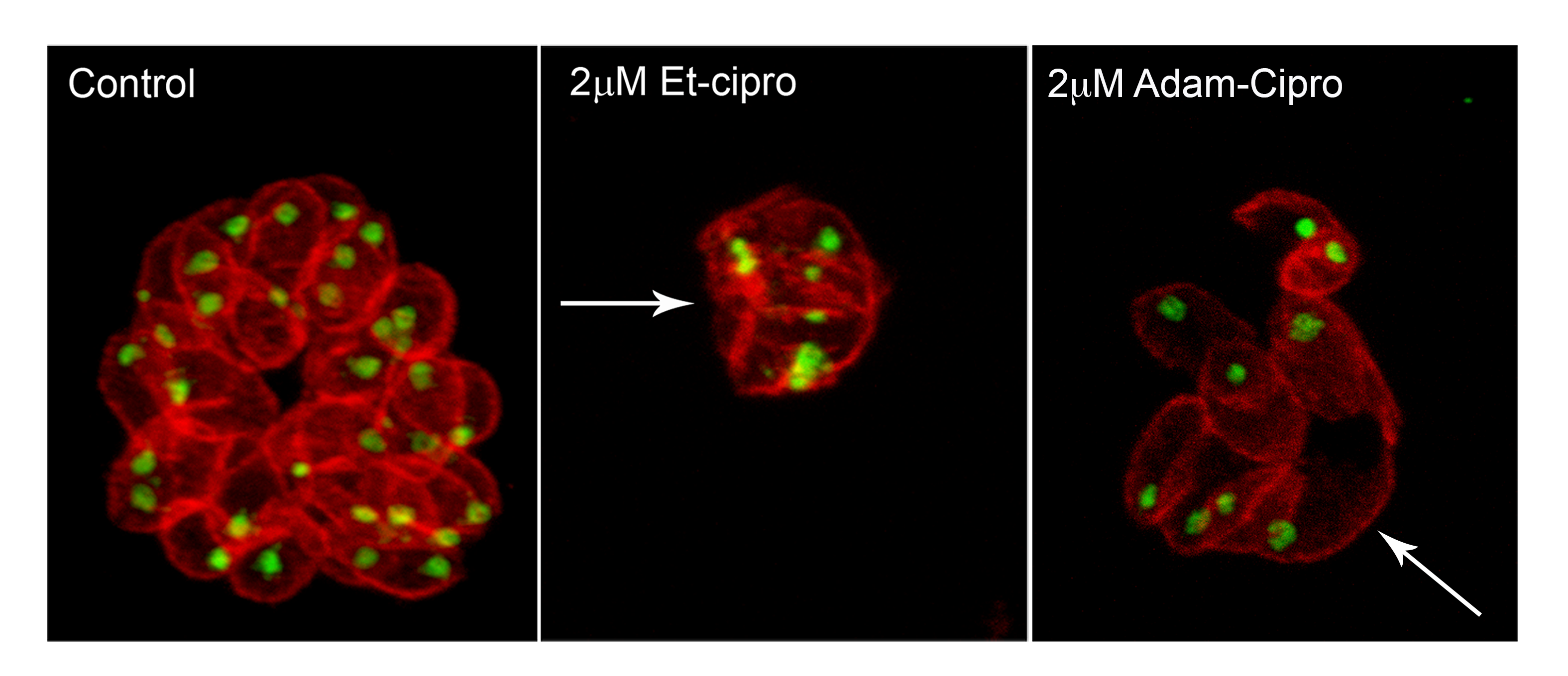

Supplement: S2 Fig — Immunoluorescence microscopy of tachyzoites labeled with anti-HSP60 (recognizing the apicoplast, in green) and anti-IMC1 (recognizing the inner membrane complex or IMC, in red) antibodies, and treated with Cipro derivatives for 6 h and then observed and kept in culture for a further 18h in the absence of drugs. Parasite division defects were observed (arrows) as a result of Cipro derivative treatment in the first event of tachyzoite division inside host cells. Even after drug removal from the medium, cell division continued to be affected. Images represent maximum projection of optical slices. (TIF) [file pone.0125705.s002.tif]
